# Driving factors of energy related CO_2_ emissions at a regional level in the residential sector of Iran

**DOI:** 10.1038/s41598-023-44975-x

**Published:** 2023-10-16

**Authors:** Behnam Ata, Parisa Pakrooh, János Pénzes

**Affiliations:** 1 Department of Social Geography and Regional Development Planning, University of Debrecen, Hungary Debrecen, 4032 Egyetem tér 1,; 2 Marie Sklodowska-Curie Postdoctoral Fellowship, Fondazione Eni Enrico Mattei (FEEM), Milan, Italy

**Keywords:** Environmental social sciences, Carbon capture and storage, Fossil fuels

## Abstract

Iran has increased its CO_2_ emissions significantly during the past few decades. The household sector in Iran contributes one of the largest sectors of CO_2_ emissions. Despite this significant contribution, the existing policies have predominantly concentrated on large-scale initiatives while overlooking the regional role in shaping and implementing these plans. Therefore, this study investigates the relationship between CO_2_ emissions and the efficient factors in three major groups including energy, climate, and household socio-economic factors. This study aims to address regional carbon emissions and develop CO_2_ reduction policies tailored to each region's specific circumstances. It focuses on planning strategies at the regional level to effectively tackle CO_2_ emissions. Household panel data of 28 provinces of Iran are employed by using both static and dynamic panel models for the years 2001 to 2019. Static estimation includes Fixed Effect (FE), Random Effect (RE) and pooled Partial least squares (PLS), Dynamic estimation includes difference Generalized Method of Moments (GMM) and system Generalized Method of Moments (GMM). The empirical result of the static method showed positive dependence of household CO_2_ emissions on Heating Degree Days (HDD), Cooling Degree Days (CDD), precipitation level, oil consumption, gas consumption, household income, size of household, and also building stocks. In more detail, educational rate, dummy variable (removal of energy subsidy), and oil price reveal the greatest negative impact on the emissions with elasticities of − 0.428, − 0.31, and − 0.15; It represents 1% increase causes − 0.428, − 0.31, − 0.15, decrease CO_2_ emissions, respectively. however, household size, gas consumption, and oil consumption show the most significant positive effects on CO_2_ emissions with 1 percent increase causes CO_2_ emissions increases by 0.1, 0.044, and 0.026, respectively. Regarding the impact of climate factors, a 1% increase in Heating Degree Days, Cooling Degree Days, and precipitation level causes CO_2_ emissions increase by 0.024%, 0.004%, and 0.011% respectively, due to an increase in fossil energy demand. Results of the dynamic method of the system Generalized Method of Moments are similar to the static estimation results, except for that household size and urbanization are not significant. Also, removing the energy subsidy for fossil fuels due to substantial subsidy in fossil fuels in Iran or implementing a re-pricing energy policy can be a beneficial way to control carbon emissions from households within the provinces of the country. However, it is important to consider that this shift could potentially transfer subsidies to investments in the private sector for renewable energies.

## Introduction

Many nations have taken specific steps to reduce their CO_2_ emissions because climate change, which is influenced by anthropogenic CO_2_ emissions, has emerged as a significant obstacle to human sustainable development^1^. Studying CO_2_ emissions is important since they are at the forefront of the current conversation about environmental protection and sustainable development^2^. Due to significant increases in greenhouse gas (GHG) emissions over the last few decades, environmental pollution has emerged as one of the most critical worldwide problems^3^. It is obvious that any discussion of changes in CO_2_ emissions over the past few decades cannot be separated from a discourse of economic changes, which include the level (or lack thereof) of economic growth, the sectors where this growth occurred, changes in incomes, and changes in how these are distributed among the population of the country^4^.

The top ten GHG polluters globally are the United States, China, India, Russia, Japan, Germany, South Korea, Canada, Iran, and the United Kingdom. Iran has had the largest increase in CO_2_ emissions among the top 10 emitters during the past 40 years—nearly 500%. Residential direct CO_2_ emissions are on the rise as well; in 2011, these exceeded 105Mt, a figure 245% more than it was in 1990^5^. Global energy consumption is anticipated to make up for all of the ground lost in 2020 as a result of the pandemic in 2021. Sharp price increases for gas, coal, and electricity have been a result of the ensuing increase in demand for all types of fuels and technology. This is obscuring indicators of deeper fundamental shifts, such as the steadily accelerating growth of renewable energy sources and electric automobiles. Experts expect to detect the second-largest increase in CO_2_ emissions in history in 2021^6^.

Greatest effort has been put into measuring the rebound for home end users in terms of increases in fuel efficiency for space heating, space cooling, and personal vehicular transportation. However, choosing the right activity metric is a major problem for these home end applications^7^. The main cause of CO_2_ emissions is regarded to be energy usage^8^. The usage of fossil fuels has a harmful effect on the environment, causing CO_2_ emissions, meanwhile modern energy options like liquefied petroleum gas (LPG) and renewable, environmentally friendly energy sources are difficult to reach in developing countries. The growing concern over the usage of fossil fuels and the withdrawal of fuel subsidies has diverted attention away from issues impacting many low-income nations, which would suffer acute political and social repercussions from the quick removal of fuel subsidies^9^.

Iran is the largest holder of global oil and gas reserves, with 158.4 billion barrels of oil and 33.5 trillion cubic meters of gas, making it a developing nation with rich and ample energy resources^10^. According to international statistics, final per capita energy consumption in Iran, in agriculture, households (public and commercial), transportation and industrial sectors is 3.3, 2.1, 1.5 and 1.6 times more than the global average, respectively^11^. Households sector is the second-largest source after transportation having produced 23.4% of all CO_2_ emissions. Therefore, about one-fourth of Iran's CO_2_ emissions come from domestic sources^12^.

The experience of European nations with developing laws and state regulatory structures for energy transfer should be given special consideration. An action plan known as the "Green Deal" was developed, a quota trading system was implemented, environmental taxes were increased, a cross-border tax was implemented, and much more in order to make the world completely hydrocarbon-neutral by the year 2050^13^. Therefore, by taking into account a variety of contributing elements including renewable energy, technical innovation, export quality, and economic growth, lowering CO_2_ emissions as a way to create a green and sustainable earth has become a desirable goal for modern scholars^14^. The link between economic growth and energy consumption is affected by several factors, including the price of energy supplies. Also, there are various events, such as revolution, war, economic sanctions, oil shocks, etc. Price is one of the most important parameters in energy demand and consumption^11^. Remarkably, Iran has been perceived for the past 60 years as a country with a rapid trend of urbanization, expanding from 31% in 1956 to 75% in 2018^15^. However, since the government pays subsidies for energy usage and has had complete control over energy prices since 1980, Iran's energy pricing mechanism is still out of step with global prices. As a result, Iranian energy prices are generally lower than global standards^16^. Accordingly, the increasing amount of CO_2_ emissions along with growth in population and GDP will increase the concentration of pollutant emissions in the household sector of Iran’s provinces^17^. With regards to CO_2_ emissions, Iran is ranked among the top ten. Due to social-political changes and industrialization, it is experiencing a rapid urbanization rate^18,19^. Iran's energy intensity based on the primary energy supply and final energy consumption according to the internal information of energy balance sheet in 2019, was 0.34 and 0.22 the barrel equivalent of crude oil in million Rials, which has increased 16.3 and 13.6 percentage, respectively compared to the previous year^11^. The choices that people make regarding household expenses and carbon dioxide emissions depend on a number of variables, including income, size, household age, and composition. In order to measure each of these elements' relative impact on household CO_2_ emissions, a comprehensive method is therefore needed to address their impact^20^.

Regional studies appear to be important in order to identify the elements that contribute to CO_2_ emissions in light of the various characteristics of the provinces in Iran, including their geographic situation, economy, social, climate, and natural and human resources^17^. In this regard, based on variables, first, employment rate and incomes are highest in industrial provinces, such as Tehran, Mashhad, Isfahan, Markazi, West Azerbaijan and Bushehr, meanwhile, in provinces such as Sistan and Baluchestan, Chaharmahal and Bakhtiari, and Khozestan are lowest. Second, education rate in provinces with above average rate of urbanization is high. In contrast, provinces with low level of urbanization are the lowest. Third, number of building stock in provinces with high population and density, such as Tehran, Khorasan, West Azerbaijan and Isfahan is higher than in provinces with smaller number of population. Forth, household sizes are highest in certain provinces such as Sistan and Baluchestan, Ilam, Kohgiluyeh and Boyer-Ahmad due to cultural characteristics. Meanwhile, in northern provinces and Tehran household sizes are the smallest. Fifth, climate factors are one of the important factors and it is completely different at regional level in Iran, some provinces like southern have totally warm weather and low precipitation in all the year including Khuzestan, Bushehr and Hormozgan which demand more electricity, however, in general most part of Iran has a four season with hot summers and cold winters. Additionally, some provinces have extreme cold weather in winter and roughly comfort summers like Ardebil, Azerbaijan, Zanjan, Hamedan, Ilam and Chaharmahal and Bakhtiari. The most precipitation is in northern provinces such as Mazandaran and Gilan. Central provinces such as Kerman, Yazd and Sistan and Baluchestan have lowest precipitation. Some provinces based on population and high rate of urbanization require more energy and emit more CO_2_, meanwhile, some provinces with lack of infrastructure and comfort climate do not demand more energy. These disparities in diverse factors among different provinces of Iran have led us to choose this study. Policies regarding regional CO_2_ emissions reduction require regional studies on factors that influence CO_2_ emissions. For instance, dry provinces may demand more electricity and different materials for buildings compared to cold provinces. Moreover, the education rate significantly varies across provinces of Iran, and increased awareness about the importance of saving energy can be another important factor leading to reduced CO_2_ emissions. Therefore, it is necessary to determine the factors that influence CO_2_ emissions on a regional scale. This will help policymakers formulate effective plans and policies to reduce CO_2_ emissions according to each province's unique situation. By identifying the significant variables that impact CO_2_ emissions, we can work towards improving these factors through appropriate policies.

The major contributions of this study are: first, this is the first and distinctive study in the literature that investigates the comprehensive elements of CO_2_ emissions at the regional level among 28 provinces of Iran. Second, this study has used the latest published and complete data over the period of 19 years (2001–2019). Third, the robust results are realized by adopting various well-equipped econometric techniques i.e. Kao test, Static estimation including Fixed Effect (FE), Random Effect (RE) and pooled (PLS), Dynamic estimation includes difference GMM and system GMM. Fourth, detailed policy suggestions, based on the findings, are provided for the researchers and policy makers to reduce CO_2_ emissions based on driving factors for environmental sustainability. Therefore, the aim of this study is to evaluate the factors influence CO_2_ emissions in the residential sector using static and dynamic models.

Firstly, the choice of Iran as the focal point of this research is substantiated by compelling reasons. Iran ranks among the top CO_2_-emitting countries globally, and its substantial fossil fuel consumption underscores the urgency of understanding the drivers of emissions within the country. Furthermore, Iran exhibits pronounced regional disparities in a range of natural and social factors, necessitating region-specific strategies for CO_2_ emissions reduction. This geographical diversity within Iran motivated our study to delve into the complexities of regional emissions dynamics. Regarding the selection of variables, our approach is multidimensional. We encompass climate variables as they play a pivotal role in influencing energy demand and emissions patterns. Additionally, we incorporate economic variables, such as energy prices, to investigate their impact on consumption behavior. Furthermore, social factors, including household size and education rates, are included due to their potential influence on emissions patterns. Notably, we introduce a critical variable—the removal of subsidies for fossil fuels—reflecting the dynamic nature of policy interventions. The major innovation of our work lies in three key aspects. Firstly, our study pioneers the examination of regional-level CO_2_ emissions dynamics in Iran, utilizing time series data, thus providing a nuanced understanding of emissions determinants at this localized scale. Secondly, our research adopts a multifaceted approach, encompassing a diverse set of variables, including climate factors, which have received limited attention at the regional level. Lastly, our study bridges the gap between localized, regional emissions dynamics and the broader national context, acknowledging Iran's role in the global climate change landscape. By considering factors that operate from the local to the national level, our research contributes a holistic perspective that has been hitherto unexplored. In summary, this study aspires to elucidate the multifaceted nature of CO_2_ emissions at the regional level within Iran, driven by the recognition of its status as a significant emitter and the existence of regional disparities. By incorporating various variables and considering Iran's climate context, we endeavor to make a substantial contribution to the field of climate change studies, with implications for policy formulation and emissions reduction strategies at both regional and national scales.

The structure of the current paper is as follows: “Literature review” section shows a literature review on the factors influencing CO_2_ emissions. "Methodology" section illustrates the study area and methodology including static and dynamic econometric models, and we introduce information on the provinces. “Results” section performs descriptive analysis and an empirical illustration of the methodology by using province data. Finally, “Conclusion and policy implications” section makes some concluding remarks, and particular policy implications are proposed.

## Literature review

The assessment of CO_2_ emissions across various sectors, including Buildings, Transport, Industrial, and Agriculture, is of paramount importance for identifying the influencing factors on CO_2_ emissions. While there have been studies concentrated on the transport sector^21^, exemplified by the establishment of a trans-log production model for Pakistan's transportation sector, incorporating labor, capital, and energy consumption as key inputs. The analysis from 1991 to 2018 indicates a consistent increase in both output and input factor elasticities, notably in the context of labour-energy and capital-energy substitutions. To optimize output and promote environmental conservation, the study proposes a strategic emphasis on augmenting capital investment and the adoption of energy-efficient technologies within the transportation industry. Furthermore, it offers policy recommendations derived from these findings.

In another study in Bangladesh^22^, it's noteworthy that labour exhibits the highest output elasticity, succeeded by energy and capital. There exists significant potential for technology-driven substitution, especially between capital and labour, as well as between capital and energy, by channelling increased capital investments into the transportation sector. This approach can facilitate the promotion of energy-efficient technologies and, consequently, the reduction of CO_2_ emissions. Furthermore, enhancements in skilled labour and the substitution of energy for labour can be achieved through strategic capital investments. However, it's essential to acknowledge that this study, while primarily focused on the building sector, a comprehensive analysis could consider multiple sectors to gain a more holistic understanding of CO_2_ emissions and multiple sectors to identify common factors influencing CO_2_ emissions.

Most studies have focused on the impact of energy consumption, incomes, temperature, urbanization and socio-economic factors like education, employment rate, etc. on CO_2_ emissions. Meanwhile, most studies believed that changes in CO_2_ emissions are driven by socio-economic, energy demand and demographic factors^23^. Effects of household consumption trends on emissions in Spain. Spanish economy's Social Accounting Matrix (SAM) that produced for 1999 used in the analysis. Combining the data on income and consumption provided by the Household Budget Continuous Survey with the final requirements of households as the exogenic account in the SAM context. In order to meet consumer demands, they analyze the pollution that is produced by both the economy and families. Also take into account how income disparity affects spending, drawing a connection between income level, consuming habits, propensity to consume, and CO_2_ emissions^2^. Economic expansion, CO_2_ emissions, and fossil fuels consumption in Iran, using the Toda-Yamamoto method, a relatively new time series methodology, for Iran from 1967 to 2007. Empirical findings point to a unidirectional Granger causation between GDP and two energy consumption proxies (consumption of petroleum products and natural gas), but no Granger causality between overall fossil fuel consumption and carbon emissions over the prolonged term. The findings as well indicate that while gas consumption promotes economic growth, carbon emissions, petroleum products, and overall fossil fuel expenditure do not.

Büchs and Schnepf^24^, they discovered that these relationships differ significantly between emission domains. While all forms of emissions increase with revenue, people with low salary, unemployed, and older families are more probable to have high home energy emissions than households of people in other groups. The study considers that these groups may be less impacted by carbon tariffs on transportation or overall emissions. This illustrates the necessity to analyze the fairness implications of mitigation plans for distinct emission domains^25^. The study investigates the regional impact of urbanization, energy intensity, energy structure, and income on HCE using a geographical weighted regression (GWR) model. The findings show that carbon emissions in the provinces have a clear spatial influence from 2000 to 2015, the effect of urbanization on household CO_2_ emissions showed an increasing trend from the southeast coast to the northwest. Although it had a negative impact in all provinces in 2005 and in certain provinces in 2010, energy intensity had a strikingly favorable impact on HCE in 2000 and 2015. In most provinces during all four years, the energy structure's elasticity coefficient on HCE was negative, meaning that increasing the use of electricity and natural gas reduced HCE. In all years, income was a significant explanatory factor for the growth in household CO_2_ emissions. The relationship between income and HCE was favorable and tended to grow over the years^26^. The STIRPAT model improved by panel estimate of the effects of characteristic domestic parameters on CO_2_ emissions from the domestic sector in China, with a focus on the three regions and the country. Location-specific effects of family size on domestic CO_2_ emissions mean that raising emissions in the eastern part will significantly reduce emissions in the middle part. While the dependent variable benefits across all locations from both energy intensity and per capita home consumption^27^. Assessment of socio-economic circumstances on CO_2_ emissions in Iran, in order to identify the important variables and their relationships to carbon emissions, a factorial design used. The findings showed that CO_2_ emissions are significantly influenced by energy consumption, including its price, non-oil GDP, citizen rate, and FDI, and that these factors and CO_2_ emissions are related linearly. The findings of the latent variable model demonstrated that, with a coefficient of 0.87, smaller energy consumption results in lower CO_2_ emissions. The findings also showed that FDI and citizen rate are the additional two extremely significant drivers, negatively and positively, respectively in increasing CO_2_ emissions through a maneuvering relationship. Energy costs and GDP from sources other than oil play a small role in explaining CO_2_ emissions^28^.

The effects of socio-economic and demographic factors on the CO_2_ emissions of homes in Thailand examined using economic input–output tables. The most important element influencing the amount of energy and CO_2_ needed is temperature (1 °C rise in temperature led to an increase of 200% in the needed energy and CO_2_). One major element, education, has a beneficial impact on direct energy and CO_2_ needs but a negative impact on secondary energy and CO_2_ needs^29^. Urbanization effects on residential CO_2_ emissions with the applied fixed effects in two stages least squares (2SLS) using an expanded Stochastic Impacts by Regression on Population Privileged circumstances and Technology (STIRPAT) model. Rising urban population proportions, GDP per capita, urban density, and overall level of urbanization are all important factors that affect residential CO_2_ emissions^30^. This study reexamines the impact of human activities on residential CO_2_ emissions in China's 28 provinces from 2000 to 2016 by dividing the country into three regions and taking regional differences into account. The primary drivers of household CO_2_ emissions are examined using an extended stochastic impact by regression on wealth, population, and technology model. Regional differences exist in China's residential CO_2_ emissions, as well as the effects of urbanization, energy use, and price elasticity. The most important variable, which favorably affects total household CO_2_ emissions is GDP per capita^31^. Personal circumstances and household energy use both have a significant impact on household carbon emissions, with different provinces and urban and rural locations experiencing the effects to different degrees. The government and society share the same objectives of reducing home carbon emissions and promoting a happy coexistence between humans and nature. In their study^32^, prediction of CO_2_ emissions in Iran created on time sequence and regression analysis, they used different regression methodologies to anticipate Iran's CO_2_ emissions in 2030 beneath the expectations of two setups. Moreover, the government does not currently have a clear program in this area. Additionally, Iran's Sixth Five-Year Development Plan lays out a number of ambitious goals, primarily in the areas of energy intensity, GDP development, and renewable energy sources, but it makes no mention of the problem of CO_2_ emissions. Results indicate that, based on the BAU's assumptions, Iran is unlikely to fulfil its obligations under the Paris Agreement, while full implementation of the ambitiously designed SDP might have achieved the goal by the end of 2018^20^. Effects of family demographics on carbon dioxide emissions, Mahabad city, Iran. Partial least squares structural equation modelling gave clear instructions on how to develop a statistical method for data analysis (PLS-SEM). According to the survey, households spend about 89.71% of their monthly energy budget on liquefied petroleum gas (LPG), 9.87% on electricity, and the remaining 0.43% on kerosene, gasoline, and diesel. Ultimately, findings of this study revealed that household age, family size, and carbon dioxide emissions—but not household education level or household income—are strongly connected with energy saving^20^.

The proposed structural model included six variables related to demographic factors, energy sources and consumptions, including educational attainment, household age, household size, income quintile, gender, and energy preservation, Partial least squares operational equation modeling presented. With the exception of academic background and income level, demographic factors and carbon dioxide emissions are highly connected with energy saving^33^. Prioritizing driving factors of domestic carbon emissions: an appliance of the LASSO model with survey data. The findings indicated that fossil fuel type and residence type can account for more than 70% of direct HCEs (Household Carbon Emissions), while income, number of residents in a town or countryside area, and energy type are the three most significant influencing factors of implicit HCEs. China will keep up its rapid urbanization and rapid consumer growth to reduce HCEs^34^. Over the past thirty years, Saudi Arabia has witnessed a significant rise in greenhouse gas (GHG) emissions. This study employs the logarithmic mean Divisia index (LMDI) method to uncover the factors influencing GHG emissions across nine sectors from 1990 to 2016. The findings highlight that the primary driver of emissions is the "energy effect," contributing 386.76 million tons of carbon dioxide equivalent (MTCO_2e_). Policymakers are urged to prioritize climate considerations in economic growth plans, potentially through transitioning to renewable energy sources, enhancing energy efficiency, and altering the energy structure to mitigate GHG emissions growth.

This paper aims to assess the impact of this electricity generation on the country's environmental quality and considers the influence of energy-efficient technological innovations^35^. The study employs structural time series modeling and logarithmic mean Divisia index (LMDI) analysis, revealing that factors like GDP, electricity generation, and population have substantial effects on carbon dioxide (CO_2_) emissions. Importantly, the findings emphasize the need for improving energy efficiency and implementing stringent environmental regulations to ensure sustainable economic growth in Saudi Arabia^17^. the researchers utilized the Theil index and Kaya factor to assess the disparity between CO_2_ emissions and energy consumption and identify the leading causes of CO_2_ emissions. The results of the disparity analysis indicated that while electricity consumption remained relatively stable, inequalities in oil and natural gas consumption increased. Furthermore, most of the disparities in energy consumption and CO_2_ emissions were found to be within-group disparities. According to the Kaya factor data, energy efficiency, with a value of 0.21, emerged as the primary driver of inequalities in CO_2_ emissions. However, the first component, energy consumption, may also contribute to inequality in the future. Alajmi^36^, highlights the significance of electricity consumption's impact on Pakistan's economy and CO_2eq_ emissions from 1990 to 2019. The study employs decomposition and decoupling methods to identify key factors influencing CO_2eq_ emissions, including population, activity, electricity intensity, and generation structure. It reveals that population growth and economic activity are major drivers of rising emissions, with weak decoupling observed^37^. This study employs ridge regression to examine the substitutability and technological progress between energy and non-energy factors. The findings suggest that various energy inputs are substitutable, particularly gas, oil, and electricity replacing coal, offering an opportunity to transition away from coal to reduce emissions. Additionally, capital exhibits superior technological progress over energy and labor, with implications for energy conservation, substitution, capital enhancement, and carbon reduction policies in emerging economies. These insights hold significance for future carbon mitigation efforts^38^. In the context of Pakistan's urbanization and industrialization, driven by fossil fuel consumption, this study examines the potential for energy and non-energy factor substitution to reduce carbon emissions. Using a translog production function and ridge regression to address multicollinearity, it analyzes output and substitution elasticities, technical progress, and carbon emission scenarios from 1986 to 2019. The findings indicate growing output elasticities, highlighting the contribution of all factors to economic growth. Certain factor pairs, such as capital-petroleum and capital-electricity, exhibit strong substitutability, driving capital growth and production. Investment scenarios suggest substantial CO_2_ emission reductions, with implications for energy conservation policies, particularly within the China–Pakistan Economic Corridor context^39^. In the context of global environmental economics and economic development, addressing environmental pollution is a critical objective. This study examines the European Union's (EU) environmental challenges resulting from its development activities. It finds that while the EU-27 increased energy consumption between 1990 and 2019, CO_2_ emissions decreased significantly. The analysis also reveals an inverted-U relationship between GDP and CO_2_ emissions, suggesting that economic growth in the EU is increasingly benefiting the environment, showcasing the effectiveness of environmental policies in promoting green growth across EU-27 countries.

This inquiry relates to policy because, if certain family types are expected to have higher emissions in some locations than in others, the distribution of mitigating measures may differ depending on the region they are applied. However, it is clear from reviewing the literature that a number of variables, such as energy consumption, energy cost, energy modes, GDP, economic growth, and the use of gas, have a significant impact on CO_2_ emissions. Most of these studies only take into account two, three, or at most four elements; they do not take into account all of the factors mentioned above simultaneously. However, research have done in regional scale are not enough and it is not considering all variables, meanwhile regional diversity including natural, and human are most important factors, and it is completely different in countries across the world. Therefore, this research is filling the previous research gap with considering comprehensive factors and at a regional scale in Iran to achieve sustainability to reduce CO_2_. Regarding the impact of CO_2_ emissions on climate change further studies are essential to considering the reasons and factors that influence CO_2_ emissions. The household sector has one of the highest CO_2_ emissions. There are some research projects performed using different methodologies to identify factors on CO_2_ emissions in the household sector. Most works show the energy consumption is one of the main reasons of CO_2_ emissions, meanwhile socio-economic and demographic factors have different effects in countries across the world, and urbanization is also another important factor in CO_2_ emissions. The innovation of this study is the research in Iran at province level covering a long period and considering various variables such as temperature, socio-economic factors, demography and energy use. The comprehensive research would identify the factors that influence CO_2_ emissions and compare the effect of certain variables together, like the effect of temperature with socio-economic factors. The methodology applied in this research, static and dynamic models, ensure comprehensive results. Finally, the present research focuses on geographical and regional differences. The study's results can help policymakers implement policies to mitigate CO_2_ emissions on a regional scale.

## Methodology

### Material

**Figure 1:**
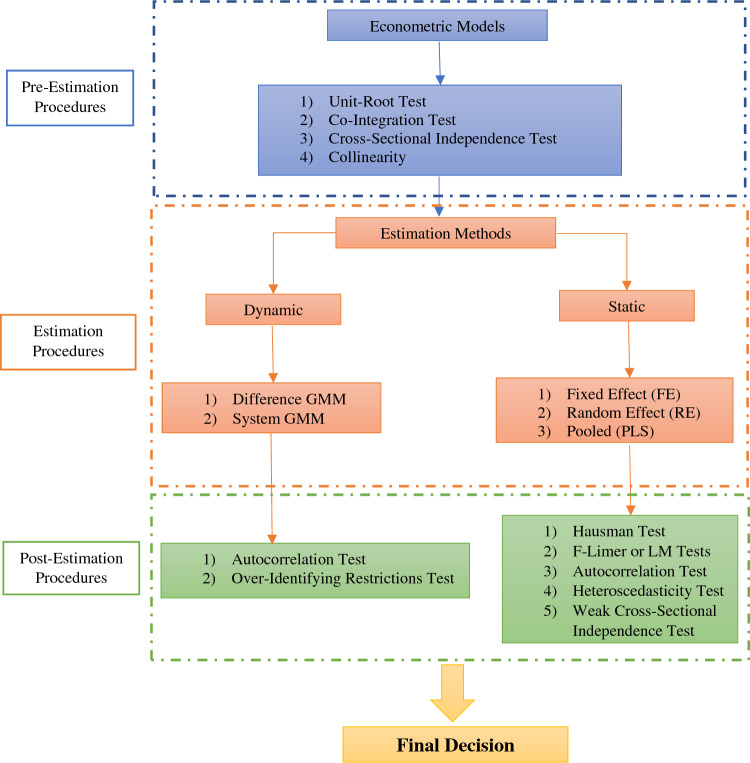
Methodology framework (Source: Authors elaborations).

To examine the causes of energy consumption and household socio-economic factors on CO_2_ emissions, a wide range of studies have used various methodologies^40–43^. Verified the impacts of economic growth, population, and energy expenditure on household CO_2_ emissions in the framework of the LMDI decomposition model. Econometric models, particularly panel and cross-sectional data analysis methods, were common general tools to study the effects of energy consumption and domestic socio-economic factors on CO_2_ emissions^1,26,28–30,44–49^. Other studies, such as^50^ used ANOVA analysis method. Likewise, we are applied panel regression to study the impacts of energy, climate, and household socio-economic factors on CO_2_ emissions of Iran’s provinces over the period between 2001 and 2019. Compared to the previous works, both static and dynamic panel regression methods are used in the study for more information and accuracy in estimation. For this purpose, a methodology framework is presented to understand the estimation procedures. According to Fig. 1, the whole process is categorized into three main steps, pre-estimation, estimation, and post-estimation procedures.

### The econometric model

To study the impacts of energy, climate, and household socio-economic factors on CO_2_ emissions, we employed both static and dynamic panel models. The specified static and dynamic equations are formulated as follows in Eqs. (1) and (2):

1 $$\begin{aligned} \mathit{log}{c}_{i,t} & ={\alpha }_{0}+{\alpha }_{1}\mathit{log}{t}_{{1}_{i,t}}+{\alpha }_{2}\mathit{log}{t}_{{2}_{i,t}}+{\alpha }_{3}\mathit{log}p{r}_{i,t}+{\alpha }_{4}\mathit{log}oi{l}_{i,t}+{\alpha }_{5}\mathit{log}ga{s}_{i,t}+{\alpha }_{6}\mathit{log}ele{c}_{i,t} \\ & \quad +{\alpha }_{7}\mathit{log}o{p}_{i,t}+{\alpha }_{8}\mathit{log}g{p}_{i,t}+{\alpha }_{9}\mathit{log}e{p}_{i,t}+{\alpha }_{10}\mathit{log}{i}_{i,t} \\ & \quad +{\alpha }_{11}\mathit{log}{s}_{i,t}+{\alpha }_{12}\mathit{log}{e}_{i,t}+{\alpha }_{13}\mathit{log}e{m}_{i,t}+{\alpha }_{14}\mathit{log}{b}_{i,t}+{\alpha }_{15}l\mathit{og}{u}_{i,t}\\ & \quad +{\alpha }_{16}{D}_{i,t}+{\alpha }_{17}{Z}_{i,t}+{\varepsilon }_{i,t} \end{aligned}$$

2 $$\begin{aligned}\mathit{log}{c}_{i,t}& ={\beta }_{0}+{\beta }_{1}l.\mathit{log}{c}_{i-p,t}+{\beta }_{2}\mathit{log}{t}_{{1}_{i,t}}+{\beta }_{3}\mathit{log}{t}_{{2}_{i,t}}+{\beta }_{4}\mathit{log}p{r}_{i,t}+{\beta }_{5}\mathit{log}oi{l}_{i,t} \\ & \quad +{\beta }_{6}\mathit{log}ga{s}_{i,t}+{\beta }_{7}\mathit{log}ele{c}_{i,t}+{\beta }_{8}\mathit{log}o{p}_{i,t}+{\beta }_{9}\mathit{log}g{p}_{i,t}+{\beta }_{10}\mathit{log}e{p}_{i,t}\\ & \quad +{\beta }_{11}\mathit{log}{i}_{i,t}+{\beta }_{12}\mathit{log}{s}_{i,t}+{\beta }_{13}\mathit{log}{e}_{i,t}+{\beta }_{14}\mathit{log}e{m}_{i,t}\\ & \quad+{\beta }_{15}\mathit{log}{b}_{i,t}+{\beta }_{16}\mathit{log}{u}_{i,t}+{\beta }_{17}{D}_{i,t}+{\upsilon }_{i,t}\end{aligned}$$

Where, *i* and *t* stand for the province and the period between 2001 and 2019, respectively. α and β are coefficients with a different type of variables, which are defined in the previous part. *Z* refers to an individual specific effect. Both *ɛ* and *ν* are error terms. *L* and *p* are lag operator and lag order, respectively.

### Econometric methodology

#### Panel unit-root test

To check the presence of unit-root in the panel data^51–57^ developed a wide series of common and individual panel unit-root tests. All of them, with the exception of Hadri’s test, are designed to test the null hypothesis of a unit-root in a panel. The alternative hypothesis is instead a controversial issue. In the study, we applied the Cross-Sectional Augmented Dickey- Fuller panel unit-root test, which was developed by^54^ to check the presence of unit-root in the individual panel data. We selected the CADF test because the test is not sensitive to the type of data set, such as balanced or unbalanced, the existence of cross-sectional dependence, and sample size. The CADF test applied to each individual series. The CADF regression is defined as Eq. (3):

3 $$\Delta Y_{i,t} = \alpha_{i} + \theta_{t}^{*} Y_{it - 1} + d_{0} \overline{Y}_{t - 1} + \sum\limits_{j = 0}^{p} {d_{j + 1} \Delta \overline{Y}_{t - j} + } \sum\limits_{j = 1}^{p} {\gamma_{ij} \Delta \overline{Y}_{it - j} + } u_{it}$$

where *Y* is the observation on the i th cross-section unit at time t. $\overline{Y }$ is the cross-section mean of *Y*, *P* stands for lag order. ${\alpha }_{i}$, ${\theta }_{t}$, ${d}_{0}$, and ${\gamma }_{ij}$ are coefficients. The null hypothesis and alternative hypothesis of the CADF unit-root test are defined^54,58^:

$$\left\{ {\begin{array}{*{20}c} {H_{0} :\theta_{t} = 0} & {\text{Null hypothesis means that all series are non-stationary processes}} \\ {H_{a} :\theta_{t} < 0} & {\text{Alternative hypothesis means that all series are stationary processes}} \\ \end{array} } \right.$$

#### Panel co-integration test

To analyze the long-term co-integration among the variables of a model, several testing procedures, such as^59–61^, are available. In the study, we applied Kao test in order to investigate the long-term co-integration among the series of both models. Kao test specifies both cross-section intercept and homogenous coefficients on the first stage regression. Under the null hypothesis, there is no long-run co-integration among the series; however, the alternative hypothesis confirms the long-term co-integration. For a regression as Eqs. (4, 5), the ADF test is as follows.

4 $$Y_{it} = \beta X_{it} + \theta Z_{it} + u_{it}$$

5 $$ADF = \frac{{t_{ADF} + \frac{{\sqrt {6N.\overset{\lower0.5em\hbox{$\smash{\scriptscriptstyle\frown}$}}{\sigma }_{v} } }}{{2\overset{\lower0.5em\hbox{$\smash{\scriptscriptstyle\frown}$}}{\sigma }_{0v} }}}}{{\sqrt {\frac{{\overset{\lower0.5em\hbox{$\smash{\scriptscriptstyle\frown}$}}{\sigma }^{2}_{0v} + 3\overset{\lower0.5em\hbox{$\smash{\scriptscriptstyle\frown}$}}{\sigma }^{2}_{v} }}{{2\overset{\lower0.5em\hbox{$\smash{\scriptscriptstyle\frown}$}}{\sigma }^{2}_{v} + 10\overset{\lower0.5em\hbox{$\smash{\scriptscriptstyle\frown}$}}{\sigma }^{2}_{0v} }}} }}$$

where, *Y*, *X*, *Z*, and *u* are the dependent variable, independent variable, intercept, and the error term, respectively. *θ* and *β* are coefficients. ${t}_{ADF}$ is the *t* statistic of *ρ* (the pair-wise correlation of the residuals). Also, ${\widehat{\sigma }}_{0v}^{2}$ and ${\widehat{\sigma }}_{v}^{2}$ stand for the long-run and simple variance of error terms^59,62^.

#### Panel cross-sectional independence test

To check the existence of cross-sectional dependence between the variables in the panel data, a Cross-Sectional Dependence (CSD) is applied. A new test for cross-sectional dependence is introduced by^54^; the test does not vary on a certain spatial weight matrix, particularly when the sample is enormous or small. Under the null hypothesis of the test, units are cross-sectionally uncorrelated, but under the alternative hypothesis, units are serially correlated. The CD statistic for the panel data model in Eq. (2) can be quantified as Eq. (6).

6 $$CD = \sqrt {\frac{2T}{{N(N - 1)}}} \left( {\sum\limits_{i = 1}^{N - 1} {\sum\limits_{j = i + 1}^{N} {\overset{\lower0.5em\hbox{$\smash{\scriptscriptstyle\frown}$}}{\rho }_{ij} } } } \right)$$

where, *ρ* is the sample estimate of the pair-wise correlation of the residuals^58,63,64^.

#### Panel collinearity test

A statistical notion known as multicollinearity describes the correlation between independent variables in a model, which makes statistical assessments less trustworthy. To estimate stable parameters in the model, we should care about multicollinearity between the variables. In other words, when there is high-level multicollinearity between variables, it will get the model more complicated to assess the connection between variables. For this, the Variable Inflation Factor (VIF) can be a useful tool to assess the severity of multicollinearity between model variables in the econometric model. In typical terms, VIF equal to 1 means that the variables are not correlated. VIF between 1 and 5 means that the variables are moderately correlated, and with VIF greater than 5, variables are extremely correlated. When VIF is more than 10, there is significant multicollinearity between variables which needs further investigation. In the current study, we used VIF for testing the multicollinearity between the variables as Eq. (7)^65,66^.

7 $$VIF = \frac{1}{{1 - R^{2} }}$$

### Estimation methods

#### Static estimation models

Panel data regression involves of two main evaluation techniques, which can be both static and dynamic estimation methods. The static panel regression types are comprised of the Fixed Effect (FE), Random Effect (RE), and Pooled (PLS) procedures. The difference among the estimation methods is in how heterogeneity has been imposed in the models. Pooled Regression (PLS) is the simple and basic estimation model with a common intercept for all panel units. In return, for both FE and RE models, heterogeneity is imposed in the form of individual-specific effects and a random factor. In more detail, FE is a regression model in which the intercept of the model can vary freely across individuals or groups and is allowed to be correlated with the dependent variable. In the case of being a very large sample (N) and short time dimensions (T), it is proper to use the FE model rather than RE. RE is a regression model in which the individual-certain effect is a random variable and is not agreed to be correlated with the independent variables. The general form of all three static panel estimation methods, PLS, FE, and RE, are formulated as Eqs. (8) to (10), respectively.

8 $$Y_{it} = \alpha + \beta X_{it} + \varepsilon_{it}$$

9 $$Y_{it} = \alpha_{i} + \beta X_{it} + \varepsilon_{it}$$

10 $$Y_{it} = \alpha + \beta X_{it} + \nu_{i} + \varepsilon_{it}$$

where *Y* and *X* are dependent and independent variables. *α* and *β* refer to coefficients. *ɛ*, *ν*, *i*, and *t* stand for error terms, random factor, province, and time period, respectively^28,67–69^.

#### Post-estimations

In order to certify the precision of the estimated model, at the primary stage, the Hausman test was employed to ascertain whether to choose the FE or RE. The null hypothesis of the test is that there is no autocorrelation between regressors and effects. Conversely, the alternative hypothesis is that there is a relationship between regressors and impacts. In other words, the null hypothesis stands with RE, and the alternative hypothesis with FE. The Hausman test is defined as Eq. (11). ${\beta }_{1}$ and ${\beta }_{0}$ are the vector of coefficients under the null and alternative hypotheses, respectively.

Then, to distinguish between the selected model in the previous step and PLS, we need to do the F-Limer or the LM (Breusch-Pagan) tests, Eqs. (12) and (13), to choose a proper model as a final decision. The F-limer test is a statistical test that is most often used for comparing statistical models, in order to identify the model that best fits the data sampled. In the test, ${R}_{FE}^{2}$ and ${R}_{RE}^{2}$ are R-squared of the FE and FE estimation models. In the case of the LM test, the null hypothesis is that the variance of the RE is zero, meaning that all the panel units have the same intercept, and we can run a PLS model regression. Conversely, the alternative hypothesis rejects zero variance for all panel units^28,68,70^.

11 $$H = (\beta_{1} - \beta_{0} )^{\prime}[{\text{var}} (\beta_{0} ) - {\text{var}} (\beta_{1} )]^{ - 1} (\beta_{1} - \beta_{0} )$$

12 $$F = \frac{{(R_{FE}^{2} - R_{RE}^{2} )(n - 1)}}{{(1 - R_{FE}^{2} )(nt - n - k)}}$$

13 $$LM = \frac{NT}{{2(T - 1)}}\left[ {\frac{{\sum\nolimits_{i = 1}^{N} {(T\,\overline{e}_{i} )^{2} } }}{{\sum\nolimits_{i = 1}^{N} {\sum\nolimits_{t = 1}^{T} {e_{it}^{2} } } }} - 1} \right]^{2}$$

Serial autocorrelation, weak cross-sectional independence, and heteroscedasticity are important issues in the error terms of panel regression models. In this regard, we applied the Wooldridge test for serial autocorrelation, the Lagrange test for heteroscedasticity, and the Pesaran test for cross-sectional independence in error terms. If results show that the model has all those problems, then the result would be unbiased and inefficient. To solve these problems, Feasible Generalized Least Square (FGLS) estimation is introduced by^71^, and the method directly takes into account all those problems in the estimation. This method allows estimation in the presence of first-order autocorrelation, heteroscedasticity, and weak cross-sectional correlation in the error terms of a panel regression model. With this method, the covariance matrix of error terms is estimated with a two-step procedure. At the primary stage, the OLS approach is applied to get the predicted residuals; then, the matrix is re-estimated by using OLS to obtain the new residuals^63,70,72–75^.

#### Dynamic estimation models

Dynamic panel data methodology is one of the most popular and helpful tools in the field of economics, particularly energy and environment analysis. Dynamic panel data model estimators have some advantages in comparison to static models, including addressing the heterogeneity of the individuals and the use of numerous instrumental variables in order to deal with the endogeneity problem. The first dynamic model estimator, which is known as the Difference Generalized Method of Moments (GMM), was introduced by^76^, and later the second estimator, the System Generalized Method of Moments (GMM), was developed by^77^. Difference GMM estimator uses the lags in differences as instruments; however, in the System GMM estimator, both lags in difference and levels are used as instruments. Both difference and system GMM estimators are designed for situations with large N and short T panels, in other words, many units and few time periods. To be precise, consider Eq. (14) a general dynamic model.

14 $$Y_{it} = \alpha + \gamma Y_{it - p} + \beta X_{it} + u_{it}$$

where, *Y* and *X* are dependent and independent variables. *α*, *β*, *γ* refer to coefficients. *ɛ*, *i*, *t*, and *p* are error terms, province, time dimension, and lag order^78–82^.

### Post-estimations

Using a large number of instruments refers to overidentification in the model. Regarding this problem, Sargan test and Hansen test, which were introduced by^83^, are used to check if the number of instrumental variables is adequate and does not cause overidentification. The Sargan test verifies the validity of the instruments in the analysis in the one-step estimations. Although, in two-step estimations, the Hansen test is recommended to check overidentification. The null hypothesis of both the Sargan and Hansen tests is that all the restrictions of overidentification are valid. However, the alternative hypothesis stands with in-valid instruments. In order to check the serial autocorrelation in the dynamic panel models, the Arellano and Bond test is suggested. The null hypothesis is that there is no autocorrelation in the model. Conversely, the alternative hypothesis stands with autocorrelation problem^78,80^.

### Data

The data used in the study are divided into two main groups, a dependent variable, CO_2_ emissions (Ton), and independent variables, which are categorized into four sub-groups, climate, energy, economic, and also social. Heating Degree Days (C^0^), Cooling Degree Days (C^0^), and precipitation (mm) are climate factors. Energy sub-group variables are named oil (Thousand Liter), natural gas (m^3^), and electricity (Kwh) consumption with their related prices (Rial per Liter, Rial per m^3^, and Rial per Kwh). Energy prices are adjusted by using the consumer price index for the fuel group (2016 = 100). We add a dummy variable with the values of 1 for the years when the removal of energy subsidy was started (2010–2019), and 0 for the rest of the period (2001–2010). Family income (Million Rial) is the only economic factor, which is adjusted by the general consumer price index (2016 = 100). Household size (person), educational rate (%), household employment rate (%), number of building stock (number of the buildings), and urbanization rate (%) are considered social factors. Data for all provinces of Iran are collected from the Statistical Yearbook of the Statistical Centre of Iran and the Energy Balance Sheets from the Ministry of Energy of Iran for the period 2001–2019 due to the availability of data. All variables are transformed into logarithmic levels to eliminate the heteroscedasticity problem and indicate the long-term elastic relationships between the variables. Table 1 shows the variables, symbols, definitions, units, and sources of all the collected data.

**Table 1:** Summary of collected data.

Variables Symbols		Definitions	Units	Sources
Dependent	Log c	Logarithm of CO_2_ Emissions	Ton	Energy Balance Sheets https://irandataportal.syr.edu/energy-environment
Independent				
(A) Climate	Log t1	Logarithm of Heating Degree Days	C^0^	Iran Meteorological Organization https://www.irimo.ir/ and power.larc.nasa.gov
	Log t2	Logarithm of Cooling Degree Days	C^0^	Iran Meteorological Organization https://www.irimo.ir/ and power.larc.nasa.gov
	Log pr	Logarithm of Precipitation	mm	Statistical Yearbook https://www.amar.org.ir/
(B) Energy	Log oil	Logarithm of Oil Products Consumption	Thousand Liter	Energy Balance Sheets https://irandataportal.syr.edu/energy-environment
	Log gas	Logarithm of Natural Gas Consumption	m^3^	Energy Balance Sheets https://irandataportal.syr.edu/energy-environment
	Log elec	Logarithm of Electricity Consumption	Kwh	Energy Balance Sheets https://irandataportal.syr.edu/energy-environment
	Log op	Logarithm of Oil Products Price	Rial per Liter	Energy Balance Sheets https://irandataportal.syr.edu/energy-environment
	Log gp	Logarithm of Natural Gas Price	Rial per m^3^	Energy Balance Sheets https://irandataportal.syr.edu/energy-environment
	Log ep	Logarithm of Electricity Price	Rial per Kwh	Energy Balance Sheets https://irandataportal.syr.edu/energy-environment
	d	Dummy Variable for Energy Subsidy		Energy Balance Sheets https://irandataportal.syr.edu/energy-environment
(C) Economic	Log i	Logarithm of Household Income	Million Rial	Statistical Yearbook https://www.amar.org.ir/
(D) Social	Log s	Logarithm of Household Size	Person	Statistical Yearbook https://www.amar.org.ir/
	Log e	Logarithm of Educational Rate	%	Statistical Yearbook https://www.amar.org.ir/
	Log em	Logarithm of Household Employment Rate	%	Statistical Yearbook https://www.amar.org.ir/
	Log b	Logarithm of Building Stock	Number of Buildings	Statistical Yearbook https://www.amar.org.ir/
	Log u	Logarithm of Urbanization Rate	%	Statistical Yearbook https://www.amar.org.ir/

### Study area

**Figure 2:**
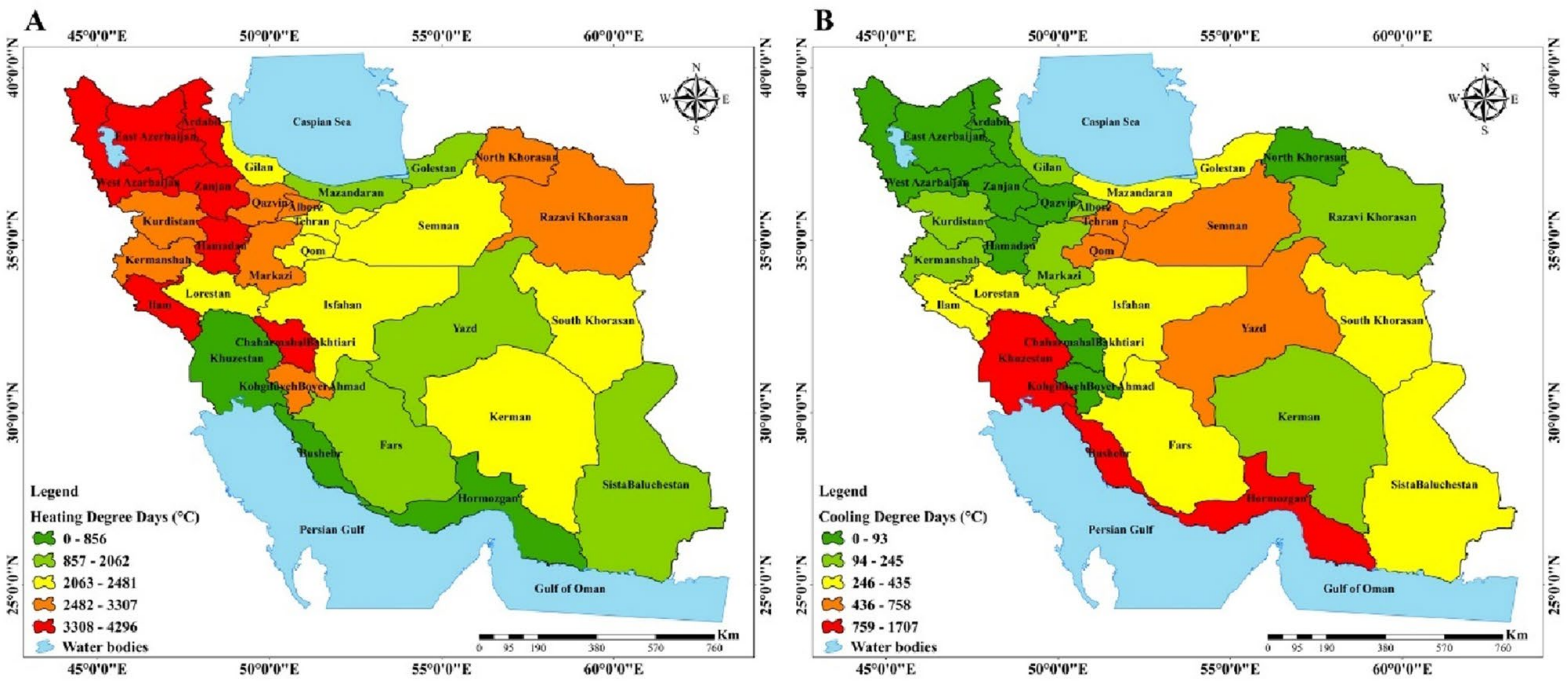
A. Average of HDD, B. Average of CDD, in the provinces of Iran from 1977 to 2019 (created by Arc GIS 10.8.2; software; https://www.esri.com/en-us/arcgis/products/arcgis-desktop/resources).

**Figure 3:**
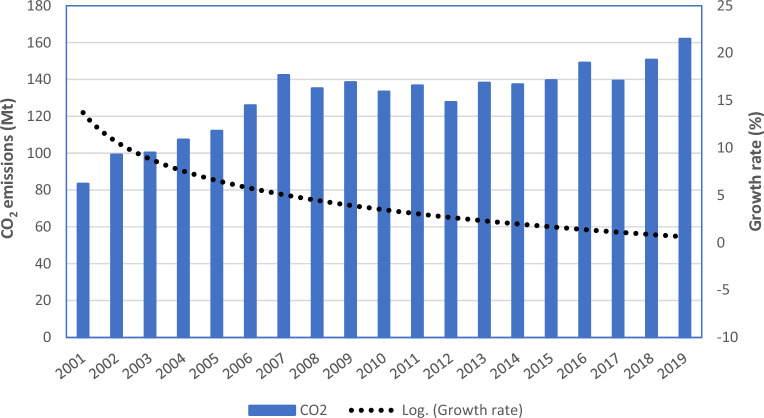
CO_2_ emissions of Iran’s residential sector (created by Microsoft excel 365; software; https://www.microsoft.com/en-us/microsoft-365/excel).

Iran is a country with climatic diversity and remarkable topography. Provinces in Iran's southern and northern Persian Gulf and Oman Sea coasts are found within one of these climatic zones. It is important to note that from the West Coast towards the East Coast, the meteorological regime changes. As shown in Fig. 2 the amount of CDD (Cooling Degree Days) is higher than HDD (Heating Degree Days) in south of Iran. Reports indicate that average amount of HDD and CDD in Khuzestan are 856 °C and 1707 °C, in Hormozgan 228 °C and 1594 °C, and in Sistan and Baluchestan 1757 °C and 435 °C, respectively. Iran's two largest Provinces, Yazd and Isfahan, experience hot, dry summers and cold, dry winters, respectively. Figure 2 shows the amount of CDD is approximately the average or slightly more than the average in Yazd and Isfahan. Meanwhile, HDD is the opposite of CDD and shows average values for Isfahan and less than the average for Yazd. provinces in the north can be classified into two categories: those near the Caspian Sea and those outside its boundaries. Golestan is situated in the southeast of the Caspian Sea, while Gilan is a northern province situated on the southwest coast. According to the long-term yearly average, the HDD of Gilan meteorological station as the representative of southwestern provinces of the Caspian Sea is 2232 °C. This HDD is decreasing towards the east, reaching 2062 °C for the Golestan. Amount of HDD for northern provinces are average which means that more heating is required than cooling during the year. One of the coldest provinces in the country's northern half, Ardebil, has cool summers and bitterly cold winters. The maps show the highest HDD among all provinces for Ardebil. The average annual temperature of its station is 14.9 °C, and its relative humidity is 71.55%. In the northeast of Iran, the Khorasan station is located which is severely affected by Siberian high pressure in the cold period of the year and experiences cold and dry conditions. The map shows high values of HDD. The Iranian stations in the west and northwest that are impacted by the Mediterranean and Red Sea systems and their rain are represented by East Azerbaijan and Kermanshah. According to the map, the amount of HDD is high for them^84^. The concentration of pollutant emissions in the household sector of Iran's provinces will rise as a result of rising CO_2_ emissions, population growth, and GDP growth^17^. Figure 3 shows the amount of CO_2_ emissions in Iran in the study period from 2001 to 2019. Amount of CO_2_ emissions increased from 82 million tons in 2001 to 162 million tons in the residential sector. Also, growth rate represents decline during the study period. Especially the trend of CO_2_ declined after 2007, and till 2013 almost no significant increase can be detected in CO_2_ emissions.

## Results

### Statistical description

Table 2 is provided to describe the statistical description of the studied variables in the natural logarithms. A balanced panel data set with a total of 532 observations was built for 28 provinces for the period from 2001 to 2019. The center of the variables distributions is measured by statistical mean, and to describe the spread of the variables, standard deviation and variance measures are reported. According to the results, most of the variables had a small standard deviation value, meaning that most of the variables indicated a small spread. In the following, the maximum and minimum values of the variables are available. Skewness and Kurtosis factors were measured to determine whether our data appeared. Most of the distributions are moderately-skewed and light-tailed relative to a normal distribution. The last column indicates the measures of the normality status of the variables, in which all the variables are strongly rejected the null hypothesis of the Shapiro–Wilk normality test. We should note that, the selected methods for the estimation do not require normally distributed data.

**Table 2:** Statistical description of the studied variables.

Variable	OBS	Mean	Std. dev.	Max	Min	Skewness	Kurtosis	Variance	Shapiro–Wilk
Log c	532	14.3	2.12	17.56	6.73	− 1.9	6.73	4.5	10.55***
Log t1	532	2.80	0.24	8.45	3.89	0.05	3	0.05	11.04***
Log t2	532	5.10	1.60	7.70	− 0.59	− 0.84	3.35	2.56	7.39***
Log pr	532	5.50	0.77	7.73	2.63	− 0.44	3.83	0.60	4.98***
Log oil	532	12.09	1.05	14.66	8.30	− 0.29	3.06	1.11	2.49***
Log gas	532	6.60	1.64	9.75	0	− 1.62	6.76	2.70	9.52***
Log elec	532	7.54	0.96	10.30	5.50	0.60	2.95	0.92	5.93***
Log op	532	6.95	0.43	7.57	6	− 0.73	2.67	0.18	7.28***
Log gp	532	6.58	0.67	7.57	5.30	− 0.05	1.73	0.45	7.60***
Log ep	532	6.22	0.21	6.49	5.76	− 0.88	2.84	0.04	8.87***
Log i	532	19.26	0.92	22.62	17.41	0.90	3.71	0.86	7.08***
Log s	532	1.34	0.15	1.91	1.02	0.71	3.20	0.02	6.21***
Log e	532	4.42	0.05	4.53	4.15	− 1.40	7.77	0.002	8.32***
Log em	532	3.23	0.23	3.77	1.85	− 1.56	8.16	0.05	8.50***
Log b	532	8.35	0.76	10.62	6.91	0.64	2.69	0.58	6.68***
Log u	532	4.17	0.18	4.56	3.75	0.20	2.51	0.03	4.26***
d	532	0.52	0.49	1	0	− 0.10	1.01	0.25	–

*Refers to 10%, ** is 5%, and *** is 1% significance level.

### Descriptive results

**Figure 4:**
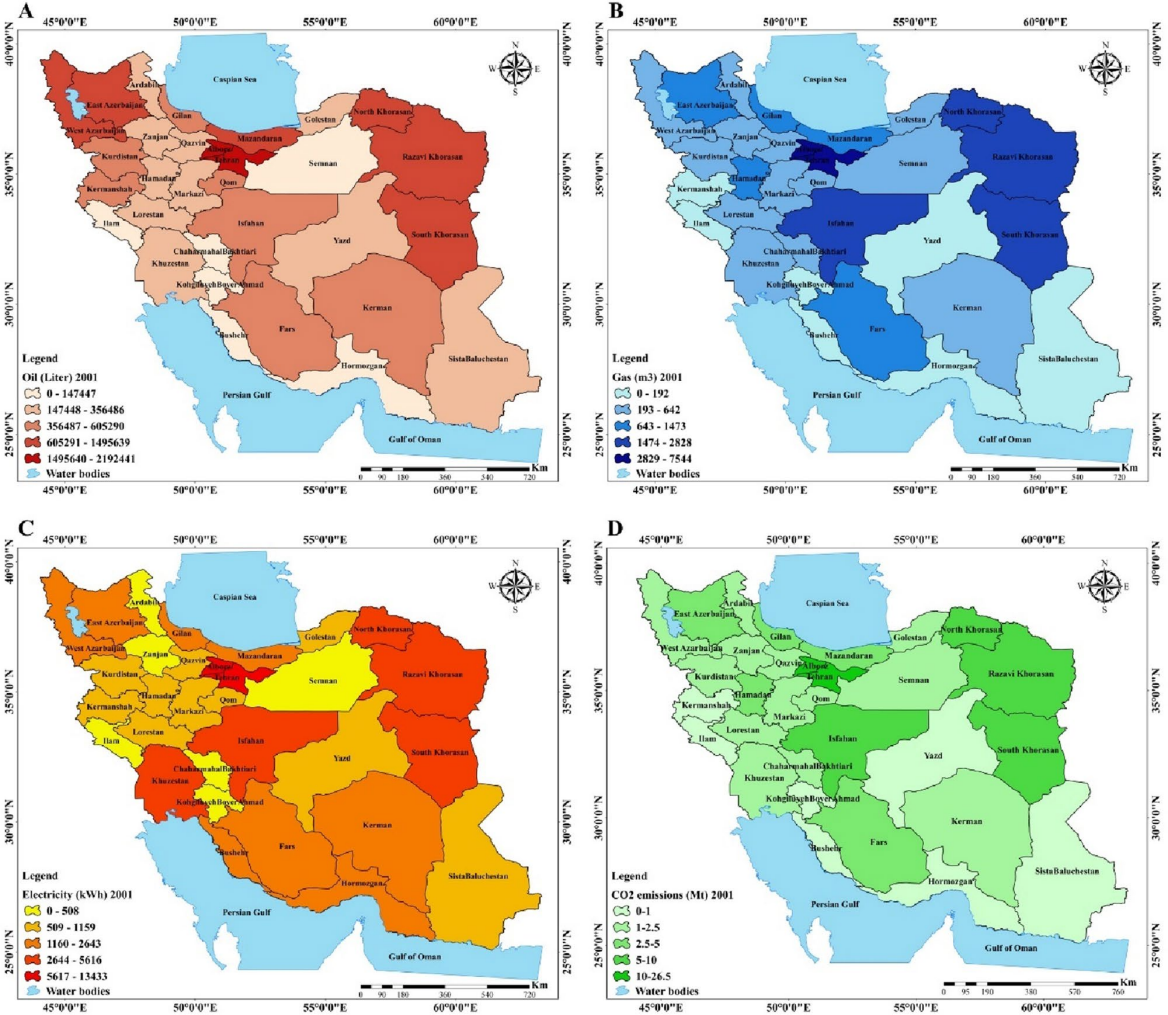
Spatial distribution maps: (**A**) oil consumption (2001), (**B**) gas consumption (2001), (**C**) electricity consumption (2001), (**D**) CO_2_ emissions (2001) (created by Arc GIS 10.8.2; software; https://www.esri.com/en-us/arcgis/products/arcgis-desktop/resources).

**Figure 5:**
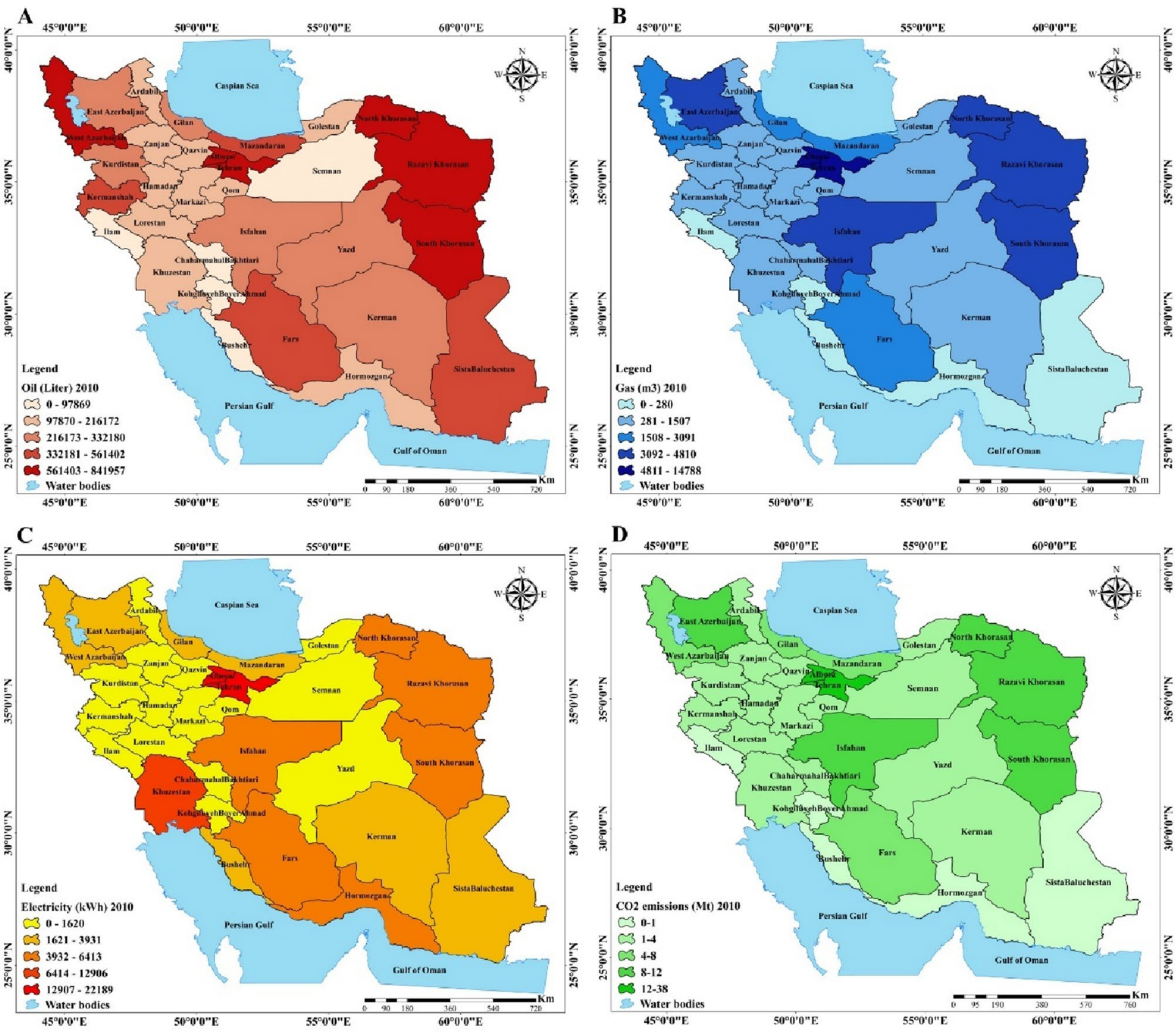
Spatial distribution maps: (**A**) oil consumption (2010), (**B**) gas consumption (2010), (**C**) electricity consumption (2010), (**D**) CO_2_ emissions (2010) (created by Arc GIS 10.8.2; software; https://www.esri.com/en-us/arcgis/products/arcgis-desktop/resources).

Due to the geography and natural, climate, cultural and economic diversity of Iran it is essential to conduct research at a regional scale. Therefore, this study investigated energy consumption and the effect of different factors on CO_2_ emissions at a regional scale. According to Fig. 4, which shows the spatial distribution of energy consumption including oil, gas and electricity in the residential sector in 2001, oil consumption in the residential sector had a particularly high proportion in Khorasan, Tehran and Azerbaijan provinces. Also, gas consumption in Khorasan, Tehran and Isfahan was extremely high, meanwhile, most of the southern provinces were without or had very low share of gas consumption. Electricity consumption had a high proportion in group 1 provinces (see Fig. 7), in contrast, Semnan, Ardebil, Zanjan, Chaharmahal and Bakhtiari and Kohgiluyeh and Boyer-Ahmad were among the provinces with very low electricity consumption. Regarding oil, gas and electricity consumption in 2010 map D of Fig. 5 shows that the share of CO_2_ emissions in group 1 provinces were so high, meanwhile, group 3 and group 4 provinces had a low share of CO_2_ emissions.

Figure 5 presents the spatial distribution of oil, gas, electricity consumption and CO_2_ emissions in the residential sector of Iran in 2010. Oil consumption in total amount dramatically declined due to switching to gas, but still the share of oil consumption among provinces like Tehran, Khorasan, East Azerbaijan and Sistan and Baluchestan were considerable. Total amount of gas consumption compared to 2001 substantially increased. Considering the increasing infrastructure in the gas sector, the share of gas in 2010 in the majority of the provinces was higher than in 2001 except for the southern provinces which still did not have access to the pipeline in the residential sector. Also, the share of electricity consumption in total amount increased. Tehran and Khuzestan had most of the electricity consumption, meanwhile, the southern provinces had the highest electricity consumption. Large cities and provinces in group 1 (see Fig. 7) still have high CO_2_ emissions.

**Figure 6:**
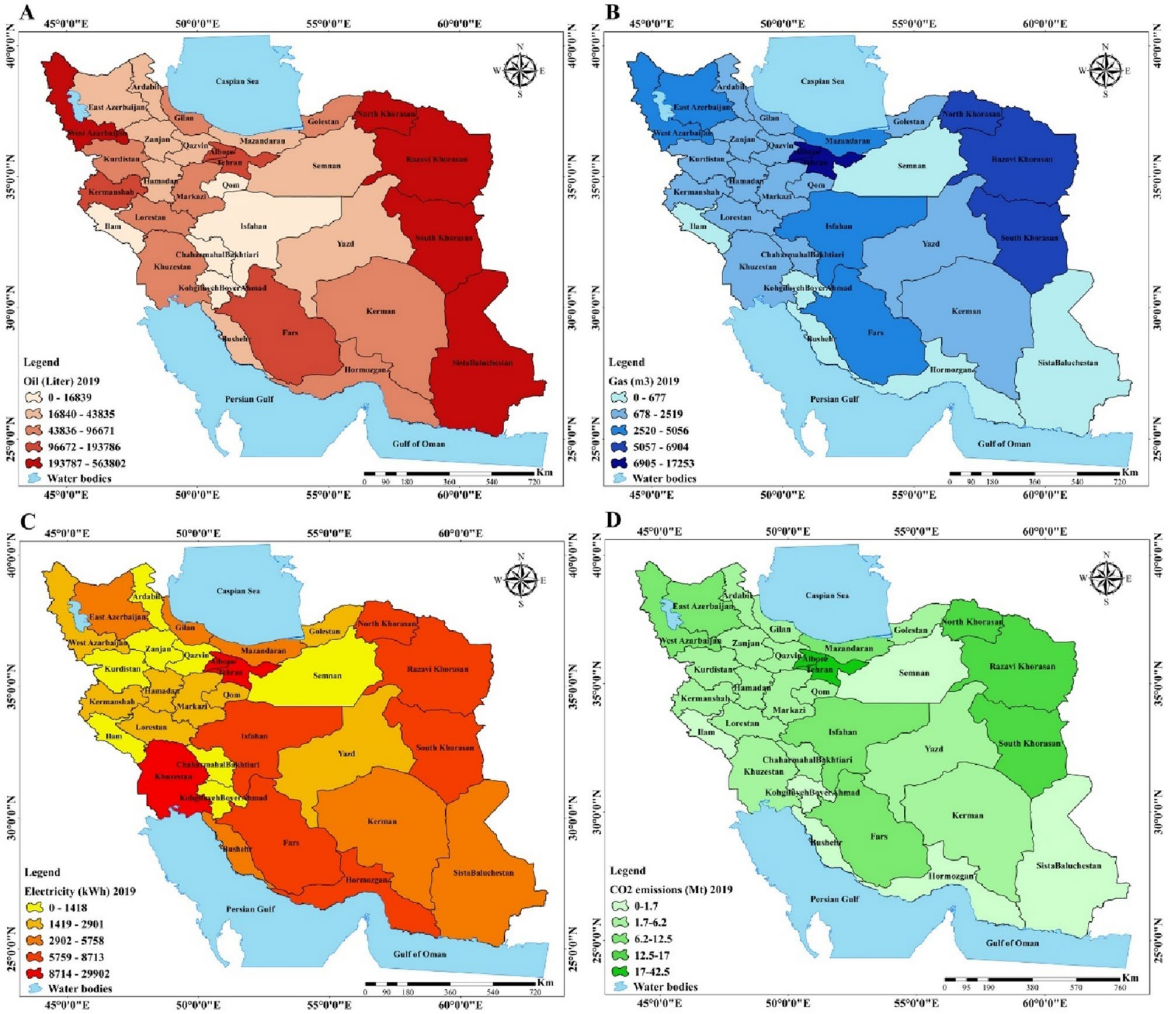
Spatial distribution maps: (**A**) oil consumption (2019), (**B**) gas consumption (2019), (**C**) electricity consumption (2019), (**D**) CO_2_ emissions (2019) (created by Arc GIS 10.8.2; software; https://www.esri.com/en-us/arcgis/products/arcgis-desktop/resources).

Figure 6 illustrates the last period of this study, 2019, when oil consumption still declined and the share of oil in the provinces which had no access to gas was high, like in Sistan and Baluchestan, Khorasan and West Azerbaijan. Compared to 2010, Tehran was not among those with the highest oil consumption due to access to gas facilities. Electricity consumption likewise in 2010 in big provinces and southern provinces had the greatest share. CO_2_ emissions increased in 2019 compared to 2010 and group 1 provinces had a high share of CO_2_ emissions. Meanwhile, group 4 provinces were among those with the lowest CO_2_ emissions.

**Figure 7:**
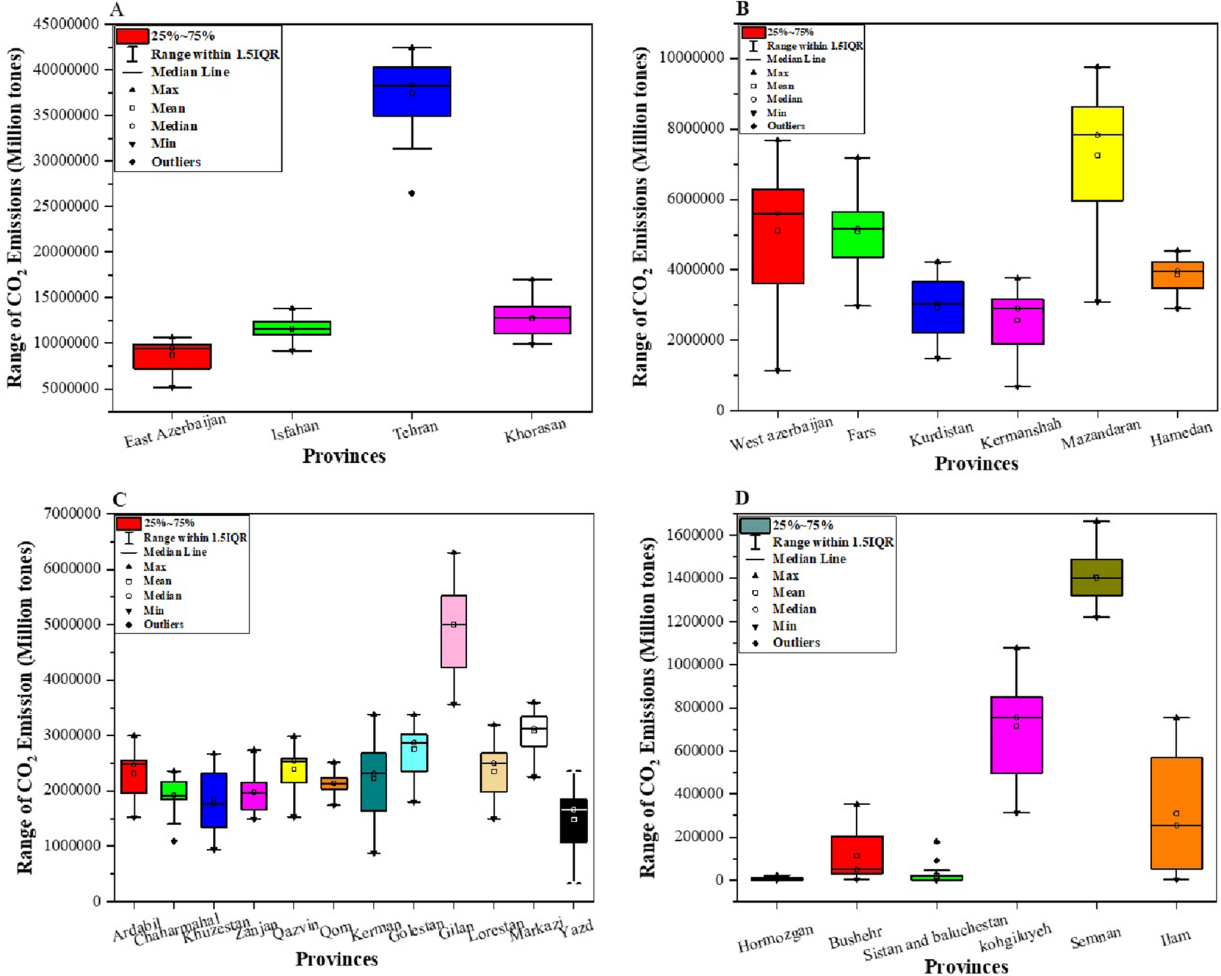
Range of CO_2_ emissions among 4 groups of provinces in Iran: (**A**) group 1, (**B**) group 2, (**C**) group 3, (**D**) group 4 (created by originlab2022; software; https://www.originlab.com).

Figure 7 shows provinces in 4 groups based on CO_2_ emissions. Group 1 (A) represents provinces with highest CO_2_ emissions. Those provinces have a large population and industrial establishments like Tehran, Isfahan, Khorasan and East Azerbaijan. Group 2 (B) include provinces with above average CO_2_ emissions including six provinces that are developing provinces. Group 3 shows average CO_2_ emissions, and this group includes the majority of provinces like Ardebil, Golestan and Yazd. Group 4 provinces have the lowest CO_2_ emissions among the provinces of Iran. Six provinces in this group are in the southern part of Iran. Therefore, regarding the range of groups in CO_2_ emissions can realize differences in CO_2_ emissions at the regional level.

### Pre-estimation results

#### Cross-sectional dependence result

The results of the Pesaran CD test for cross-sectional independence are reported in Table 3 below. According to the results, the null hypothesis of the test is significantly rejected for all variables; in other words, standard errors in the following estimates are biased. Using the Driscoll and Kray standard errors in estimations and the FGLS method are commonly accepted solutions to deal with this phenomenon^64,74^.

**Table 3:** Cross-sectional dependence results.

Variable	CD-test	P-value	Correlation
Log c	70.60***	0.00	0.83
Log t1	44.53***	0.00	0.52
Log t2	11.94***	0.00	0.14
Log pr	31.54***	0.00	0.37
Log oil	62.94***	0.00	0.74
Log gas	76.24***	0.00	0.93
Log elec	80.67***	0.00	0.95
Log op	84.75***	0.00	1.00
Log gp	84.75***	0.00	1.00
Log ep	84.75***	0.00	1.00
Log i	75.61***	0.00	0.89
Log s	83.80***	0.00	0.98
Log e	83.37***	0.00	0.98
Log em	30.12***	0.00	0.35
Log b	53.00***	0.00	0.62
Log u	68.61***	0.00	0.81
d	84.75***	0.00	1.00

*Refers to 10%, ** is 5%, and *** is 1% significance level.

#### Multicollinearity result

Regarding the multicollinearity test, see Table 4, all individual inflation variation factor (VIF) values are well below the generally accepted threshold of 10, which means that there is no multicollinearity among independent variables. Furthermore, the average VIF is below 10, indicating that multicollinearity does not impacts our models. Therefore, we can trust the precision of the estimated coefficients and easily interpret the models.

**Table 4:** VIF values of the variables.

Variables	VIF	1/VIF	Mean VIF
Log t1	6.50	0.15	5.03
Log t2	2.66	0.37	
Log pr	1.57	0.63	
Log oil	3.95	0.25	
Log gas	6.52	0.15	
Log elec	1.49	0.67	
Log op	5.90	0.17	
Log gp	6.84	0.14	
Log ep	9.73	0.10	
Log i	7.67	0.13	
Log s	9.80	0.10	
Log e	4.65	0.21	
Log em	1.74	0.57	
Log b	4.13	0.24	
Log u	2.59	0.38	
d	4.78	0.20	

#### Panel unit-root result

We used the Cross-Sectional Augmented Dickey- Fuller panel unit-root test, which was developed by^54^ to test all the variables for the stationary. Table 5 reveals the results of the tests in two different ways, with and without trends. According to the results, all variables, except for precipitation level, gas and electricity consumption, and household income, are nonstationary in the specification without trend. When a trend is included, we get different results, CO_2_ emissions, precipitation level, gas and electricity consumption, and building stock are stationary at their levels, while the remaining variables are not. After taking the first difference, all the nonstationary variables were stationary at 1% significance level (shown in the last column).

**Table 5:** CADF Unit-root test result.

Variable	Lags	Without trend		With trend		Result
		Z [t-bar]	P-value	Z [t-bar]	P-value	
Log c	1	2.28	0.98	− 1.85**	0.03	I(0)
Log t1	1	4.53	1.00	2.96	0.99	I(1)
Log t2	1	0.45	0.67	− 0.83	0.20	I(1)
Log pr	1	− 3.64***	0.00	− 1.91**	0.02	I(0)
Log oil	1	− 0.92	0.17	0.24	0.60	I(1)
Log gas	1	− 2.35***	0.00	4.01***	0.00	I(0)
Log elec	1	− 3.33***	0.00	− 2.61***	0.00	I(0)
Log op	1	22.56	1.00	20.90	1.00	I(1)
Log gp	1	22.56	1.00	20.90	1.00	I(1)
Log ep	1	22.56	1.00	20.90	1.00	I(1)
Log i	1	− 3.93***	0.00	− 0.83	0.20	I(0)
Log s	1	1.85	0.96	1.78	0.196	I(1)
Log e	1	3.25	0.99	3.63	1.00	I(1)
Log em	1	− 0.29	0.38	− 1.17	0.12	I(1)
Log b	1	− 3.78***	0.00	− 1.40*	0.08	I(0)
Log u	1	10.70	1.00	9.12	1.00	I(1)

*Refers to 10%, ** is 5%, and *** is 1% significance level.

#### Panel co-integration result

When some variables are nonstationary, we need to test whether they are co-integrated. For this aim, the relationship between the dependent and independent variables is further identified by using the Kao co-integration test. According to the results in Table 6, all five variants of the Kao co-integration test strongly rejected the null hypothesis of no co-integration. The presence of co-integration means that the estimation results are not superiors, and there is a long-time relationship among the variables.

**Table 6:** Kao co-integration test results.

Modified Dickey-Fuller t		Dickey-Fuller t		Augmented Dickey-Fuller t		Unadjusted Modified Dickey-Fuller t		Unadjusted Dickey-Fuller t	
Statistic	p-value	Statistic	P-value	Statistic	P-value	Statistic	P-value	Statistic	P-value
− 6.31***	0.00	− 12.6***	0.00	− 7.44***	0.00	− 15.8***	0.00	− 15.9***	0.00

*Refers to 10%, ** is 5%, and *** is 1% significance level.

### Estimation results

#### Static estimation

We estimated the impacts of energy consumption, climate, and household socio-economic factors on CO_2_ emissions by using different panel data regression methods. Firstly, the Hausman test was applied. According to the results in Table 8, the null hypothesis of the test is strongly rejected, meaning that the Fixed Effect (FE) model is a consistent model for estimations. Secondly, the F-test is carried out to select the final model between FE and PLS models. The test is statistically significant at the 1% significance level, and the FE model is selected for further investigations (see Table 7). Thirdly, the Wooldridge test is employed to examine autocorrelation. With the test results in Table 8, the null hypothesis of the test is clearly rejected; in other words, there exists the autocorrelation problem in the estimated FE model. In the following, the Wald test is used to examine group-wise heteroscedasticity in the model. And based on the result of the Wald test in Table 7, the heteroscedasticity problem exists in the estimated FE model. Finally, the Pesaran cross-sectional dependence test is employed to examine the correlation between panel units. Based on the test results in Table 7, the null hypothesis of the test is rejected, and cross-sectional dependence exists in the estimated model. Based on our findings from the tests in Table 7, the estimation results of the FE model can be misleading and biased. Next, to solve those problems, we applied the FGLS estimation method, which generates reliable and precise results (see Table 8 below).

**Table 7:** Model selection tests.

Tests	Statistics	P-value
Hausman (between FE and RE)	252.91***	0.00
F-Limer (between FE and PLS)	18.21***	0.00
Autocorrelation (Wooldridge)	47.26***	0.00
Heteroscedasticity (Wald)	38.29*	0.09
Weak cross-sectional dependence (Pesaran)	54.57***	0.00
Final model	FGLS	

*Refers to 10%, ** is 5%, and *** is 1% significance level.

**Table 8:** FGLS estimation results.

Variables	Coefficients	Std. dev.	P-value
Log t1	0.024***	0.007	0.00
Log t2	0.004**	0.002	0.03
Log pr	0.011***	0.003	0.00
Log oil	0.026***	0.004	0.00
Log gas	0.044***	0.003	0.00
Log elec	− 0.09***	0.012	0.00
Log op	− 0.15***	0.026	0.00
Log gp	− 0.026**	0.013	0.04
Log ep	− 0.038	0.030	0.20
Log i	0.011**	0.006	0.05
Log s	0.11***	0.015	0.00
Log e	− 0.428***	0.030	0.00
Log em	− 0.022**	0.011	0.05
Log b	0.014***	0.005	0.00
Log u	− 0.067***	0.019	0.00
d	− 0.31***	0.022	0.00
Wald test	4.46 × 107***		0.00

*Refers to 10%, ** is 5%, and *** is 1% significance level.

The empirical results of the FGLS estimation are shown in Table 8. All variables, with the exception of electricity price, are statistically significant at 1% and 5% significance levels. Results show a positive dependence of household CO_2_ emissions on HDD, CDD, precipitation level, oil consumption, gas consumption, household income, size of household, and also building stocks; in other words, household CO_2_ emissions grow as these factors increase. Conversely, electricity consumption, oil and gas prices, educational rate, household employment rate, urbanization rate, and dummy variable reveal a negative relationship with the emissions, which means that household CO_2_ emissions decrease as these factors increase. In more detail, educational rate, dummy variable, and oil price reveal the greatest negative impact on the emissions with elasticities of − 0.428, − 0.31, and − 0.15, respectively. In contrast, household size, gas consumption, and oil consumption show the most significant positive effects on CO_2_ emissions with elasticities of 0.1, 0.044, and 0.026, respectively. Regarding the impact of climate factors, 1% increase in Heating Degree Days (HDD), Cooling Degree Days (CDD), and precipitation level, causes increasing CO_2_ emissions by 0.024%, 0.004%, and 0.011%, respectively. Regarding the energy factors, we found that the higher oil and gas consuming provinces, including Tehran, East Azerbaijan, Khorasan, Isfahan, Mazandaran, and West Azerbaijan, produce higher CO_2_ emissions. 0.026% and 0.044% increases in CO_2_ emissions are due to a 1% increase in oil and gas consumption like it was found by^27,31^. Meanwhile, Lotfalipour et al.^2^ found no Granger causality between total fossil fuel consumption and carbon emissions over the long term. Figure 4 illustrates that big provinces have produced most CO_2_ which was also increasing in northern and western provinces during the study period. The coefficient for electricity consumption is negative. This implies that 1% increase in electricity consumption, leads to a decrease in CO_2_ emissions. As we expected, 1% increase in energy prices for oil and gas causes preferable decrease in CO_2_ emissions. The dummy variable for removing energy subsidies showed a negative effect on CO_2_ emissions in the western provinces during the study period. We found the same results in the impacts of energy prices on the emissions. This implies that higher energy prices support the environment. These latter results similar to the findings of^16,30,85^. Meanwhile^27^ demonstrated that raising the price of energy will not result in a reduction in CO_2_ emissions. Six social and economic coefficients in the estimated model, household income, household size, household employment, educational rate, building stock, and urbanization rate, influenced positively and negatively CO_2_ emissions. This implies that the higher the income and members of households in Iran’s provinces, the higher the CO_2_ emissions like Miao et al.^30^ found. In other words, households tend to increase CO_2_ emissions through different factors like an increase in energy consumption, as their income increases (a variant of the EKC theory). An increase of one person in household size will cause an increase of 0.11% in household CO_2_ emissions. Also, 1% higher level of education and employment rate decrease CO_2_ emissions by 0.428% and 0.022%, respectively like^28^ found. A decrease of 0.067 in CO_2_ emissions is a result of an increase of 1% in urbanity like it was found by^29,30,86^. For building stock, we expected a negative effect, but the results show that the higher the number of the buildings is, the higher the CO_2_ emissions will be with 0.014 elasticities. In other words, building operations in energy use within the provinces of Iran are responsible for the environmental degradation.

### Dynamic estimation

Table 9 shows the result of the Hausman test to select between the system GMM and the difference GMM estimators. The null hypothesis is that the preferred model is the difference GMM and the alternative hypothesis stands with the system GMM. Based on the results, the null hypothesis is strongly rejected, and the system GMM results are selected for further analysis. It is worth to note that the results of the difference GMM estimator are similar to the system GMM estimator. This implies the robustness of the estimated models across different dynamic methods.

**Table 9:** Hausman test for model selection.

Test	Result
Hausman test (between difference and system GMM)	2626.6***

*Refers to 10%, ** is 5%, and *** is 1% significance level.

The findings of the system GMM estimator are presented in Table 10, which are similar to the results of the previous section. The results of the Sargan test show that the null hypothesis of the test is not rejected. The instruments are valid and uncorrelated with the error terms. The null hypothesis of the Arellano-Bond tests is not rejected; it means that there is no autocorrelation problem in the estimated model. Based on the estimated results, we find that all the independent variables, except for household size and urbanization, are statistically significant, at least at the 10% level. Among the estimated variables, HDD, CDD, precipitation level, oil and gas consumption, household income, building stock, and the dummy variable show a positive effect on households CO_2_ emissions. However, the sign of the coefficients for energy prices, educational rate, employment rate, and dummy variable are negative; it means that those variables have a negative impact on households CO_2_ emissions. The greatest negative and positive impacts are shown in the educational rate and gas consumption, with elasticities of − 0.38 and 0.89. In other words, social and energy factors show a considerable effect on household carbon emissions.

**Table 10:** System GMM results.

Variables	Coefficients	Std. dev.	P-value
l.Log c	0.046***	0.019	0.00
Log t1	0.038***	0.008	0.00
Log t2	0.007***	0.002	0.00
Log pr	0.015***	0.003	0.00
Log oil	0.023***	0.006	0.00
Log gas	0.89***	0.016	0.00
Log elec	− 0.07***	0.016	0.78
Log op	− 0.10***	0.030	0.00
Log gp	− 0.074***	0.006	0.00
Log ep	− 0.15***	0.032	0.00
Log i	0.049***	0.006	0.00
Log s	0.17	0.14	0.21
Log e	− 0.38***	0.19	0.00
Log em	− 0.064**	0.033	0.05
Log b	0.019***	0.004	0.00
Log u	− 0.38	0.44	0.38
d	− 0.31***	0.04	0.00
Sargan test	25.28		1.00
Bond test AR(2)	1.00		0.31
Wald test	5.62 × 106***		0.00

*Refers to 10%, ** is 5%, and *** is 1% significance level.

In the case of energy factors, an increase of 1% in oil and gas consumption leads to an increase in CO_2_ emissions by 0.023% and 0.89%, respectively. Wang et al.^25^, in contrast, found that using more natural gas decreased HCE. However, electricity energy shows a different impact on the emissions, in which a decrease of 0.07% in CO_2_ emissions is due to an increase of 1% in electricity consumption similar to the findings of^25^. Regarding energy prices, we found that 0.10%, 0.074%, and 0.15% decrease in CO_2_ emissions are caused by 1% increase in oil, gas, and electricity prices, respectively. Similar to^16,30,85^. Meanwhile is different to^27^, provided here demonstrated that raising the price of energy will not result in a reduction of CO_2_ emission. The dummy variable, as we expected, reveals a negative and significant impact on emissions. In other words, removing the energy subsidy or re-pricing policy can be a helpful way to control the carbon emissions from the household side in the provinces of the country (Tehran, Khorasan, Mazandaran). These findings are the same as those of^16^.

Household income, as an economic factor, reveals a moderate and positive elasticity. CO_2_ emissions tend to keep increasing with household income. An increase of 1% in income leads to increases in emissions by approximately 0.045%. The employment rate and the educational rate of households show a highlighted negative impact on emissions, which means that those social factors play a crucial role in household CO_2_ emissions with elasticities of − 0.38 and − 0.064. In addition, building operations within the provinces of Iran are responsible for increases in household CO_2_ emissions, in which an increase of 1% in building stock enhances CO_2_ emissions by 0.019%. Meanwhile^87,88^ found that increase in building stock mitigates CO_2_ emissions.

## Discussion

Based on this research, we applied for the first time 16 variables’ effect on CO_2_ emissions in Iran at the regional level, according to the static and dynamic model, most of the variables were significant. According to the review of the literature, some parts of our results are consistent with previous studies, and on the other hand, some of our results are differing from previous studies. A significant part of our research produces specific results in regional context that have never been determined in previous studies in Iran. In more details in the static model 0.026% and 0.044% increases in CO_2_ emissions are due to a 1% increase in oil and gas consumption like it was found by^27,31^. Meanwhile, is contrary to^2^. The dummy variable for the theoretical removing of energy subsidies showed a negative effect on CO_2_ emissions in the western provinces during the study period. We found the same results in the impacts of energy prices on the emissions. This implies that higher energy prices support the environment. These latter results similar to the findings of^16,30,85^. Meanwhile is contrary to^27^. Higher income and members of households in Iran’s provinces, the higher the CO_2_ emissions like^35,36,89^ found. Meanwhile is contrary to^39^. An increase of one person in household size will cause an increase of 0.11% in household CO_2_ emissions. Also, 1% higher level of education and employment rate decrease CO_2_ emissions by 0.428% and 0.022%, respectively like^28^ found. A decrease of 0.067 in CO_2_ emissions is a result of an increase of 1% in urbanity like it was found by^29,30,86^. For building stock, we expected a negative effect, but the results show that the higher the number of the buildings is, the higher the CO_2_ emissions will be with 0.014 elasticities. In other words, building operations in energy use within the provinces of Iran are responsible for the environmental degradation.

Among the climate factors, HDD shows the greatest positive effect on CO_2_ emissions the results are very similar to those of^90^. In the case of energy factors, an increase of 1% in oil and gas consumption leads to an increase in CO_2_ emissions by 0.023% and 0.89%, respectively. in contrast with Wang et al. (2018). However, electricity energy shows a different impact on the emissions, in which a decrease of 0.07% in CO_2_ emissions is due to an increase of 1% in electricity consumption similar to the findings of^25^. Regarding energy prices, we found that 0.10%, 0.074%, and 0.15% decrease in CO_2_ emissions are caused by 1% increase in oil, gas, and electricity prices, respectively. Similar to^16,30,85^. Meanwhile is different to^27^. The dummy variable, as we expected, reveals a negative and significant impact on emissions. In other words, removing the energy subsidy or re-pricing policy can be a helpful way to control the carbon emissions from the household side in the provinces of the country (Tehran, Khorasan, Mazandaran). These findings are the same as those of^16^. Household income, as an economic factor, reveals a moderate and positive elasticity. CO_2_ emissions tend to keep increasing with household income. An increase of 1% in income leads to increases in emissions by approximately 0.045%. The employment rate and the educational rate of households show a highlighted negative impact on emissions, which means that those social factors play a crucial role in household CO_2_ emissions with elasticities of − 0.38 and − 0.064. In addition, building operations within the provinces of Iran are responsible for increases in household CO_2_ emissions, in which an increase of 1% in building stock enhances CO_2_ emissions by 0.019%. Meanwhile in contrast with^87,88^.

Most of the missing parts in research about related energy CO_2_ emissions is a regional and spatial part. Because most of the researchers as engineers work on technical energy issues and mostly neglect regional and spatial planning factors. But it is for the first time at a regional scale in Iran, therefore, most findings are novel and practical for policymakers, government, and companies related to renewable energies. In more detail, based on the descriptive section and empirical section, each one of the variables influences in a different way at the regional scale, factors effect in western provinces are completely different in eastern, even some provinces close to each other show different behaviour.

## Conclusion and policy implications

The study employed panel data using static and dynamic methods in provinces of Iran from 2001 to 2019 to investigate factors influencing CO_2_ emissions at a regional scale in Iran. Based on the results, the factors influencing CO_2_ emissions include energy consumption, climate, and socio-economic impacts. Among the factors, energy consumption and climate (HDD, CDD) had the greatest influence on CO_2_ emissions, in more detail, provinces with high populations and more energy use including Tehran, Khorasan, Isfahan, and East Azerbaijan were the highest CO_2_ emitters. Meanwhile, the impact of temperature on CO_2_ emissions was in different ways among provinces, such provinces like Ardebil, East and West Azerbaijan, Zanjan, Hamedan, Ilam and Chaharmahal and Bakhtiari with the highest HDD, which more CO_2_ emissions based on the results. In contrast, provinces like Khuzestan, Bushehr, Hormozgan, and Yazd with the highest CDD, demand more electricity and less CO_2_ emissions according to results, our findings are in line with^90^ and^25^. Obviously, Iran is a country with vast resources of fossil fuels and ranks among the top 10 countries in CO_2_ emissions. Therefore, it is necessary to implement reforms in various sectors, particularly in the household sector, as it is one of the highest CO_2_ emitters. Mitigating CO_2_ emissions in this sector would contribute to a transitional trajectory, reducing CO_2_ emissions and decreasing dependency on fossil fuels. Comparing different factors regarding their effects on the CO_2_ emissions of each region yields invaluable information for the government and policymakers to identify the factors and create a region-specific plan to avoid wasting investments. Also, certain factor pairs, such as capital-petroleum and capital-electricity, exhibit strong substitutability, driving capital growth and production. Investment scenarios suggest substantial CO_2_ emission reductions, with implications for energy conservation policies, particularly within the China–Pakistan Economic Corridor context^38^. Additionally, capital exhibits superior technological progress over energy and labour, with implications for energy conservation, substitution, capital enhancement, and carbon reduction policies in emerging economies. These insights hold significance for future carbon mitigation efforts^38^.

During the study period, the southern provinces of Iran, such as Sistan and Baluchestan, Hormozgan, and Bushehr, exhibited the lowest CO_2_ emissions. This can be attributed to the lack of access to gas infrastructure in these provinces. However, their high temperatures throughout the year led to generates fewer CO_2_ emissions, thus verifying the results. According to the estimation results, energy use and temperature are among the most influential factors, with provinces experiencing high heating degree days (HDD) exhibiting greater CO_2_ emissions. Additionally, populous provinces with high consumption rates, such as Tehran, Isfahan, and Khorasan, also have higher CO_2_ emissions.

Regarding the results among variables, such as education rate, oil price, and the dummy variable (indicating the removal of subsidies for fossil fuels), household size emerged as the most influential factor in static estimation. Our findings in education rate are in line with^28^ and in household size and income with^34,38,89^. This finding suggests that socio-demographic and energy-related factors have a greater impact than other variables on CO_2_ emissions. Consequently, it becomes imperative to enhance awareness about the culture of energy consumption among people. In the transition to a Net Zero Emissions (NZE) scenario compared to the Alternative Policy Scenario (APS) by 2030, changes in behaviour are responsible for approximately 25% of the additional direct emissions reductions in the buildings sector. Additionally, these behavioural changes lead to a decrease in the sector's electricity consumption and indirect emissions, contributing to more than 3% of the overall reductions in indirect emissions from buildings between the APS and NZE. These changes include adjusting heating and air conditioning settings, opting for line drying, and reducing water heating temperatures. It's worth noting that achieving these savings does not necessitate the adoption of new technologies or investments but does require heightened consumer awareness and engagement^91^. Increasing education on energy-saving practices and promoting renewable energies in the residential sector, in parallel with the gradual removal of fossil fuel subsidies, should be central to the government's policy focus. In line with the dummy variables and oil price analysis, the gradual removal of oil subsidies can be a strategic policy approach. Based on the dynamic estimation results, it is evident that gas consumption holds significant sway over CO_2_ emissions. Thus, a similar plan to gradually reduce gas subsidies would be prudent. According to the education variable, the importance of raising the awareness of families about saving in energy consumption in Iran was one of the most important issues which was proved by both models. The building stock variable shows that with the increasing proportion of new buildings energy consumption does not decrease which indicates that new buildings are not using standard material and insulation. Therefore, CO_2_ emissions are increasing. Consequently, the government should make new directives and rules about new buildings based on energy conservation.

Building materials and conditions are crucial factors in achieving energy efficiency and reducing CO_2_ emissions. Therefore, implementing regional planning based on the climate situation of each province can significantly improve energy efficiency in buildings. For instance, traditional Persian architectural practices in provinces like Isfahan, Yazd, and Kerman involve the use of wind towers with mud brick materials, enabling sustainable and natural ventilation in buildings, eliminating the need for air conditioning (AC). By embracing these climate-appropriate building techniques on a larger scale and incorporating them into new construction projects, it becomes possible to reduce the reliance on electricity for cooling purposes. As a result, the overall demand for energy, particularly electricity, in these provinces can be notably decreased. This approach not only contributes to environmental conservation and the mitigation of CO_2_ emissions but also offers economic benefits. Reduced dependence on electricity for cooling leads to lower energy consumption, which can translate into cost savings for both individual households and businesses. Also, heat pumps represent the most significant potential for electrification within the buildings industry, replacing the use of fossil fuel boilers for heating purposes^91^. In conclusion, this research emphasizes the significance of reforming energy policies at a regional scale, tailored to harness the unique potential of each province. By understanding the varying factors affecting CO_2_ emissions in different regions, the government and policymakers can formulate targeted and effective strategies to achieve a sustainable and greener future for Iran (Supplementary Information).

### Supplementary Information

Supplementary Information.

## Data Availability

The datasets generated and/or analysed during the current study are available in the (Ata et al.^17^). https://drive.google.com/drive/folders/1VjWS_RGVKXB_W4cZaXSz2lAsdmR7kH1K?usp=sharing.
